# Progress in Treatment of Stricture of Urethra

**Published:** 1880-12

**Authors:** 


PROGRESS IN THE TREATMENT OF STRICTURE OF THE URETHRA.

Some remarks were made on this subject by Sir H. Thompson, at the annual meeting of the British Medical Association, in Cambridge, August, 1880. As illustrations of this advance during the last thirty years in England, the doctor mentioned five points.

1. A general recognition of the principle that a delicate and gentle manipulation of any instruments in the urethra is alone trustworthy or permissible, in the place of that which was formerly greatly prevalent, viz., that urethral obstruction might often be overcome mainly by force.

2. The substitution of very pliable and taper intruments for silver and stiff gum-elastic instruments in much of the treatment, both in ordinary and in continuous dilatation.

3. A more general acceptance of the doctrine, that, given time, patience, and gentle handling, very few strictures should be met with which cannot be fairly and successfully traversed by an instrument passed through them into the bladder. At the same time, an undoubted improvement is to be noted in the mode of operating for those exceptional cases in which the surgeon fails to accomplish that object.

4. A more general acceptance of the doctrine that dilatation of the urethra, whether with or without incision, may be carried with advantage to a somewhat higher degree than had for some time previously been regarded as desirable.

5. The substitution of internal urethrotomy in some form for the application of caustics, and for external urethrotomy on a guide. Each of the topics named is then considered somewhat in detail. In connection with the subject of the "calibre," or "diameter" of the urethra, or the amount of its dilatability, he refers to Dr. Otis's revival of the theory of "the large diameter of the urethra." He records his sense of the value of this point, but he adds that "it is a very easy thing to damage irreparably some individuals by overdistending the urethra." Thompson also opposes another doctrine which is associated with the preceding, viz., that stricture of the urethra is permanently cured by complete division of all the diseased tissues affecting the passage. In speaking of the many methods of performing internal urethrotomy, he says that the principles which govern a sound procedure are more essential points for the surgeon to discover and to teach, than a consideration of small details. These principles he briefly states as follows: 1. The necessity for a physical examination before operating, to detect and estimate the narrowed portions of the urethra. This is best accomplished, in his opinion, by means of a series of metal bulbs on slender stems, taking care not to regard as changes of disease those points at which the urethra itself is naturally only slightly dilatable. These bulbous exploring sounds he invariably used, advocating them as essential to diagnosis, in his first work, twenty-six years ago. He still prefers them to any others, as safer, less irritating, and not less efficient than more complex instruments which have been devised. 2. The necessity for accomplishing a complete division of all the morbid tissue constituting the stricture, by an incision carried through it, no matter what part of the urethra, or how much of it, is involved in the disease. As a general rule, he thinks, this is most efficiently done by a slender blade, carried beyond the stricture and made to cut from within outward; this latter proviso being, however, an open question. The important point is that any alleviation of the patient's condition attained by operation will be transitory if any part of the narrowing be left undivided. 3. He regards it as essential, after such division, to place at once a full-sized catheter for some hours in the bladder, to ensure a free outlet for the urine, and prevent all possibility of extravasation of urine into and through the incisions thus made. 4. The necessity for passing full-sized bougies subsequently, at occasional intervals, in order to effect free distention of the walls of the urethra, which lie in almost constant opposition, and so to prevent reunion of divided surfaces by the first intention. Finally, he declares that the great desideratum of the present time unquestionably is the discovery of a mode of treatment which shall permanently restore to the strictured passage its original dilatability; and he adds that a thoughtful consideration of the pathological condition which constitutes organic stricture does not embolden him to hope that such a result can be insured by the application of any principles of action at present known to us.—*The British Medical Journal.*—*New York Medical Record.*

